# Border-Associated Macrophages in CNS Health and Disease: A Comprehensive Review of Ontogeny, Heterogeneity, and Functional Plasticity at Neural Interfaces

**DOI:** 10.1080/17590914.2026.2687503

**Published:** 2026-07-03

**Authors:** Xueting Liu, Mingyue Li, Zengrong Wei, Tong Shang, Wei Zou

**Affiliations:** a Heilongjiang University of Chinese Medicine, Harbin, China; b Acupuncture and Moxibustion Department (III), The First Affiliated Hospital of Heilongjiang University of Chinese Medicine, Harbin, China

**Keywords:** Border-associated macrophages, microglia, neuroimmunology, neuroinflammation, single-cell transcriptomics

## Abstract

Border-associated macrophages (BAMs) represent a specialized population of tissue-resident immune cells strategically positioned at the critical interfaces between the central nervous system (CNS) and peripheral circulation, including the meninges, choroid plexus, and perivascular spaces. As frontline sentinels of the neuroimmune system, BAMs perform essential functions in immune surveillance, barrier integrity maintenance, and homeostatic regulation, yet their unique biology and disease-associated roles remain incompletely characterized compared to parenchymal microglia. This review aims to synthesize current knowledge on BAM ontogenetic origins, compartment-specific heterogeneity, transcriptional programs, and functional outputs in both health and neurological disorders. We conducted a comprehensive literature analysis integrating findings from lineage tracing studies, single-cell RNA sequencing, spatial transcriptomics, and functional interrogation in animal models of disease. The results reveal that BAMs exhibit remarkable cellular diversity shaped by distinct ontogenetic origins—primarily yolk sac-derived erythro-myeloid progenitors with variable contributions from fetal liver and postnatal monocytes depending on anatomical compartment. Compartment-specific marker combinations (CD206, LYVE1, CD163, MHCII) define functionally distinct subsets, and core transcriptional regulators including PU.1 and IRF8 maintain BAM identity while CSF-1/IL-34-CSF1R signaling governs survival and renewal. In neurological disorders including ischemic stroke, Alzheimer’s disease, multiple sclerosis, and brain tumors, BAMs display pronounced double-edged roles, transitioning from protective homeostatic guardians to pathogenic drivers depending on disease stage and microenvironmental context. This comprehensive analysis establishes a unified framework for understanding BAM biology and identifies critical opportunities for developing subset-specific therapeutic strategies targeting these interface macrophages in neurological diseases.

## Graphical Abstract

**Figure:**
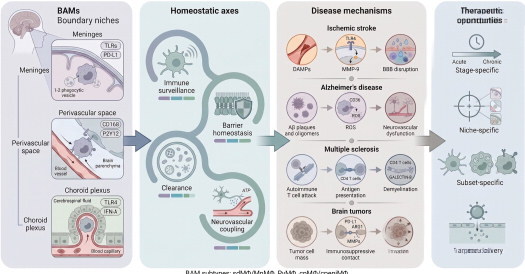

## Introduction

Macrophages are effector cells of the innate immune system and were originally described as cellular “sensors and repairers” in response to infection and tissue injury, exhibiting pronounced pleiotropic functions in initiating inflammation, regulating the immune response, and maintaining tissue homeostasis (Davies et al., 2013; Lazarov et al., 2023). They are widely distributed across virtually all tissues and organs, including the central nervous system (CNS), and can be broadly classified into tissue-resident macrophages and monocyte-derived macrophages (MDMs) (Lazarov et al., 2023). Macrophages originating from distinct developmental sources display marked differences in spatial localization, developmental trajectories, and immunological properties, while also exhibiting substantial phenotypic heterogeneity and functional plasticity, with their activation states and effector functions being finely tuned by local microenvironmental cues (Lazarov et al., 2023, Shapouri-Moghaddam et al., 2018).

Within the brain immune system, in addition to parenchymal microglia, there exists a distinct population of non-parenchymal brain macrophages located at the brain-circulation interface, collectively referred to as border-associated macrophages (BAMs) (Penati et al., 2025). In the literature, the terms central nervous system-associated macrophages (CAMs) and BAMs are often used synonymously to describe macrophages residing outside the parenchyma at CNS interface compartments; in this review, the term BAMs is used consistently to avoid conceptual ambiguity. The concept of the “brain border” emphasizes the long-term residence of BAMs within specialized interface compartments, including the choroid plexus, meninges, and perivascular spaces, each representing anatomically and functionally distinct microenvironments (Utz et al., 2020). These interfaces serve not only as critical portals for information exchange between the brain and the peripheral immune system but also as niches enriched with diverse immune cell subsets, within which BAMs can be further subdivided into subdural macrophages (sdMΦ), meningeal macrophages (MnMΦ), perivascular macrophages (PvMΦ), stromal choroid plexus macrophages (cpMΦ), and choroid plexus epithelial macrophages (cpepiMΦ) (Dermitzakis et al., 2023; Patel et al., 2025). Each subset is characterized by distinct molecular signatures and functional specializations, collectively contributing to immune surveillance, maintenance of barrier integrity, and dynamic regulation of the central immune microenvironment (Dalmau Gasull et al., 2024).

It is important to emphasize that BAMs, parenchymal microglia, and MDMs infiltrating under pathological conditions have frequently been subject to conceptual overlap or even interchange in the literature due to differences in marker selection and definitional criteria, thereby representing a major source of interpretative discrepancies and conflicting conclusions in the field (Penati et al., 2025). Although both BAMs and microglia are developmentally linked to yolk sac (YS)-derived primitive macrophage lineages, they differ in key aspects of ontogenetic complexity and maintenance strategies in adulthood: in mature organisms, BAMs residing in regions such as the dura mater and choroid plexus can be partially replenished by bone marrow-derived monocytes (Goldmann et al., 2016; Van Hove et al., 2019), whereas microglia generally retain an embryonic origin throughout the lifespan and rely predominantly on local self-renewal. In parallel, under pathological conditions, peripheral monocytes infiltrate the CNS and differentiate into MDMs, which may overlap with BAMs in morphology and certain molecular features, thereby further complicating the attribution of cellular identity, origin, and function (Penati et al., 2025). Therefore, when evaluating the physiological and pathological roles of BAMs, clearly defining their spatial compartments, dynamic origins, and marker systems, while rigorously distinguishing them from microglia and MDMs, represents a prerequisite for improving cross-study comparability and fostering consensus.

Based on this background, this review centers on the interface localization and cellular identity of BAMs: it first outlines the ontogenetic origins and biological characteristics of BAMs across distinct compartments, then summarizes their marker genes and transcriptomic heterogeneity, and delineates the key regulatory networks that preserve cellular identity and drive functional state transitions. Building on this framework, the review further integrates the specialized roles of BAMs in CNS homeostasis, with particular emphasis on their double-edged effects and potential mechanisms in diseases such as ischemic stroke (IS), Alzheimer’s disease (AD), multiple sclerosis (MS), and brain tumors, and concludes by synthesizing emerging research trends and critical open questions in the field, to provide a systematic reference for understanding brain border immune regulation and exploring potential therapeutic strategies.

Several recent reviews have summarized BAM/CAM ontogeny, marker systems, homeostatic functions, and disease involvement from complementary perspectives, including stroke-focused BAM biology, CNS-associated macrophage niches, brain-resident macrophage plasticity, and BAMs as gatekeepers of brain homeostasis and immunity (Dalmau Gasull et al., 2024; Fliegauf et al., 2026, Gerganova et al., 2022; Sun & Jiang, 2024; Van Hove et al. 2025; Vara-Pérez & Movahedi, 2025; Zhao et al., 2026). Building on these studies, the present review aims to provide an updated and integrative framework in four respects. First, it organizes BAM biology around a tri-interface logic linking the meninges, perivascular spaces, and choroid plexus to subset-specific functions. Second, it explicitly distinguishes BAMs from parenchymal microglia and infiltrating MDMs while clarifying that BAMs and CAMs are used as synonymous interface macrophage terms. Third, it incorporates emerging brain-border structures, including arachnoid cuff exit (ACE) points, dural-associated lymphoid tissue (DALT), the subarachnoid lymphatic-like membrane (SLYM), and skull-dural channels, as anatomical contexts that may reshape BAM niches and neuroimmune communication. Fourth, it integrates newly published disease-related findings in AD, IS, TBI, and glioblastoma/radionecrosis to emphasize how BAM functions shift according to disease stage, spatial compartment, and microenvironmental stress.

## Ontogenetic Origin and Biological Characteristics of BAMs

### Ontogenetic Origin of BAMs

In contrast to the relatively uniform and stable embryonic origin of parenchymal microglia, the ontogenetic origin of BAMs exhibits a dynamic pattern characterized by predominant early seeding followed by variable supplementation at later stages (Prinz et al., 2021). During early mouse embryogenesis (embryonic day [E] 7.5–8.5), the YS generates erythro-myeloid progenitors (EMPs) through endothelial-to-hematopoietic differentiation (Stremmel et al., 2018). EMPs progressively transition from moderately immature CD45^+^ c-kit^lo^ CX3CR1^-^ A1 precursors into CD45^+^ c-kit^-^ CX3CR1^+^ A2 macrophage progenitors, which subsequently migrate via the developing vasculature and enter CNS border-associated structures between E9.5 and E10.5, giving rise to the earliest cohort of BAM precursors (Kierdorf et al., 2013; Prinz et al., 2021). Within this developmental window, two transcriptionally distinct primitive macrophage populations can already be identified in both the YS and brain tissues: one population preferentially expresses BAM-associated genes such as Lyve1, Ms4a4a, and CD206, whereas the other shows higher expression of microglia-associated genes, including Sall1, Hexb, and P2ry12. Notably, these divergent trajectories retain relatively stable phenotypic distinctions even at late embryonic stages (e.g., E18.5), indicating that although BAMs and microglia share a common primitive macrophage lineage, their fate specification and microenvironmental imprinting begin to diverge during embryogenesis (Brioschi et al., 2023; Utz et al., 2020). These early CNS-infiltrating progenitors predominantly give rise to MnMΦ and contribute to a subset of PvMΦ, while retaining long-term self-renewal and population maintenance capacity, thereby establishing a cellular foundation for the stable presence of BAMs in adulthood (Goldmann et al., 2016; Utz et al., 2020; Van Hove et al., 2019).

In addition to YS-derived EMPs, progenitors originating from the fetal liver have also been implicated in the establishment of BAMs, particularly in relation to the formation of specific compartments (Sun & Jiang, 2024). After E10.5 in mice, the fetal liver progressively becomes the primary hematopoietic organ during embryogenesis and generates monocyte/myeloid precursors; accumulating evidence and recent reviews generally support the notion that subsequent fetal hematopoietic waves contribute, at least in part, to the establishment and replenishment of the CNS border macrophage pool, with a relatively more pronounced contribution to choroid plexus-associated macrophages (Du et al., 2026; Sun & Jiang, 2024). Therefore, the ontogenetic origin of BAMs does not follow a single linear lineage but instead reflects the combined influence of “programmed embryonic seeding” and “fetal liver-derived supplementation,” which are subsequently shaped by tissue-specific microenvironmental selection pressures to generate compartment-specific population architectures.

Upon entry into adulthood, the BAM population is maintained predominantly through local clonal expansion and self-renewal; however, this principle is not uniform across CNS border compartments (Goldmann et al., 2016; Lee et al., 2021; Sun & Jiang, 2024). In regions characterized by higher barrier permeability and closer interaction with the peripheral hematopoietic system, such as the dura mater and the choroid plexus stroma, BAMs are more susceptible to continuous peripheral replenishment or partial replacement, whereas in more protected microenvironments, embryonically derived components remain comparatively stable (Sun & Jiang, 2024; Utz et al., 2020). When CNS homeostasis is disrupted, these compartmental differences become further amplified: physical damage to vascular or barrier structures, as well as neuroinflammatory stimuli, markedly enhances the infiltration of peripheral immune cells into brain border regions, thereby altering the cellular origin and turnover dynamics of BAMs (Pedragosa et al., 2018; Sun & Jiang, 2024; Wang et al., 2024). *In vivo* two-photon imaging and multilinage tracing studies have demonstrated that CCR2^+^Ly6C^high^ monocytes represent a major source of BAM replenishment following perturbation of adult CNS homeostasis; upon entry into CNS border compartments, these short-lived monocytes can differentiate into longer-lived BAM-like cells and integrate into preexisting networks, indicating a dual maintenance mechanism characterized by predominant self-renewal coupled with adaptable peripheral supplementation (Wang et al., 2024). This feature further distinguishes BAMs from microglia, which typically rely almost exclusively on the self-renewal of embryonically derived cells throughout the lifespan, with minimal contribution from circulating monocytes (Ginhoux & Guilliams, 2016; Prinz et al., 2021).

**Figure 1.**
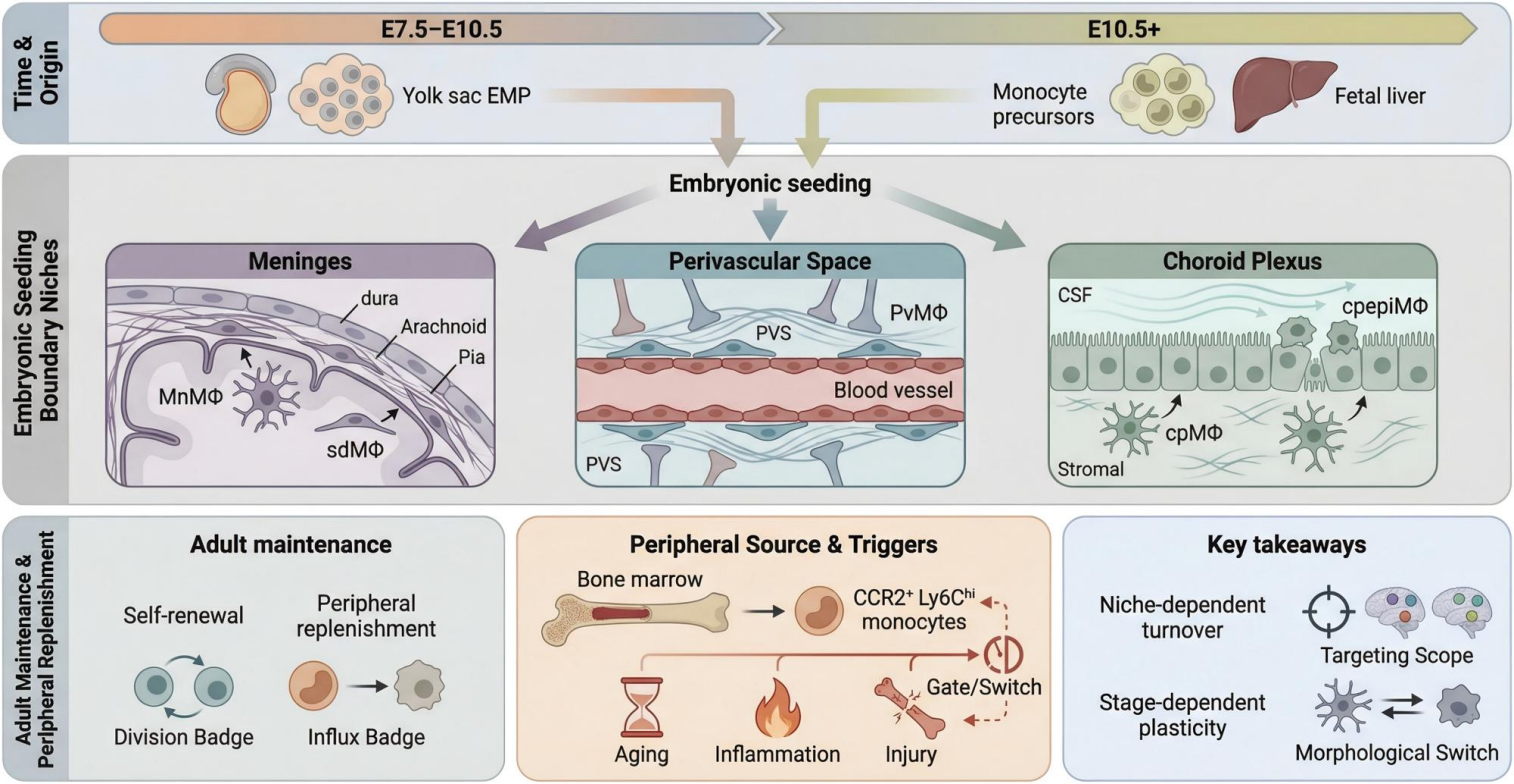
Schematic overview of the ontogenetic origins and maintenance mechanisms of BAM subsets, including subdural macrophages (sdMΦ), meningeal macrophages (MnMΦ), perivascular macrophages (PvMΦ), stromal choroid plexus macrophages (cpMΦ), and choroid plexus epithelial macrophages (cpepiMΦ).

It should be noted that the current evidence on the ontogenetic origin of BAMs remains limited, particularly due to the lack of fully unified phenotypic definitions across studies. For example, some investigations have defined BAMs primarily as CD206^+^ cells, whereas others have favored markers such as LYVE1^+^, and these differences in classification criteria can lead to inconsistencies in source inference and functional attribution when comparing distinct developmental time windows, anatomical compartments, or disease models (Sun & Jiang, 2024; Utz et al., 2020; Wu et al., 2021). Consequently, before further delineating the dynamic origins of BAMs and clarifying their boundaries with microglia and MDMs, establishing a more standardized, cross-study-comparable identification framework remains a critical prerequisite for building consensus in the field and supporting subsequent mechanistic investigations and translational exploration (Figure 1).

### Biological Morphology and Spatial Niches

Distinct BAM subsets exhibit pronounced morphological heterogeneity, which is closely associated with the spatial architecture and barrier properties of the brain border compartments they inhabit (Dalmau Gasull et al., 2024; Penati et al., 2025). In general, macrophage morphology tends to correlate with functional state: under homeostatic conditions, surveillance-oriented phenotypes typically display abundant cellular processes that expand surface area and enhance the sensing of local signals and humoral components, whereas exposure to inflammatory or injurious stimuli promotes a transition toward an ameboid morphology that facilitates migration and phagocytic clearance (Kierdorf et al., 2019). Although the direct causal relationship between morphology and functional output requires further *in vivo* validation, the concept that niche constraints establish a morphological baseline while morphological plasticity enables rapid responsiveness has emerged as a useful framework for understanding interface-specific functional specialization of BAMs.

Within the meningeal compartment, spatial positioning determines both the nature of incoming signals and the interface-related tasks assigned to resident cells. sdMΦ located in the dura mater reside in close proximity to the skull bone marrow, a highly permeable and immunologically active structure, rendering them particularly receptive to peripheral immune inputs and well suited for barrier defense (Cugurra et al., 2021; Mazzitelli et al., 2022). Imaging and histological analyses have shown that sdMΦ often exhibit elongated cell bodies with limited branching, occasionally adopting a fibroblast-like appearance, and frequently display an MHC II^+^ phenotype, consistent with enhanced antigen presentation and immune surveillance capacity (Kim et al., 2023; Rustenhoven et al., 2021). In contrast, MnMΦ located within the leptomeningeal-arachnoid layers display greater morphological diversity, commonly adopting multipolar shapes with abundant dendrite-like processes, characteristic of a surveillance-oriented tissue-resident macrophage phenotype (Hiraki & Tsuruta, 2025; Rebejac et al., 2022). Their retained motility supports patrol functions within the confined meningeal space, enabling continuous monitoring of cerebrospinal fluid (CSF) and associated pathways; under inflammatory conditions, MnMΦ undergo process elongation accompanied by cell body rounding, indicating a shift from a homeostatic surveillance state toward a phagocytic response state (Carneiro-Nascimento et al., 2025; Rebejac et al., 2022).

The perivascular compartment provides BAMs with a highly structured niche characterized by pronounced geometric constraints. PvMΦ are positioned between the glial and vascular basement membranes, align longitudinally along blood vessels, and typically exhibit spindle-shaped or band-like morphologies, with “finger-like” processes tightly apposed to the perivascular spaces, encircling the vasculature and forming close contacts with endothelial cells and pericytes (Ineichen et al., 2022; Wen et al., 2024). This intimate apposition and circumferential organization provide the structural basis for PvMΦ to monitor the blood-brain barrier (BBB) and exert localized immunoregulatory functions, while also enabling interface-related tasks, such as antigen presentation, when required (Fabriek et al., 2005; Goddery et al., 2021; He et al., 2016). Notably, canonical perivascular spaces are not fully established during early development, and ultrastructural analyses indicate that these spaces gradually emerge and expand during the postnatal period between P3 and P10 (Masuda et al., 2022), suggesting that the spatial constraints and interface contacts of PvMΦ undergo remodeling from the perinatal to juvenile stages in concert with maturation of the neurovascular unit.

The choroid plexus represents a critical gateway between the blood and CSF and constitutes a key component of the blood-CSF barrier, with its interface properties shaping distinct morphological and migratory behaviors of choroid plexus macrophages (Cui et al., 2021). By regulating CSF composition and volume and participating in the filtration of metabolic waste and potentially harmful substances from the CSF, the choroid plexus contributes fundamentally to the maintenance of brain homeostasis (Cui et al., 2021; Lun et al., 2015). Within this microenvironment, cpMΦ and cpepiMΦ display complementary spatial behaviors: cpMΦ typically exhibit relatively stationary cell bodies and are biased toward local surveillance and interface homeostasis, while their processes remain highly dynamic to support immune patrolling; in contrast, cpepiMΦ more often adopt an ameboid morphology with shorter processes and enhanced somatic motility, allowing them to patrol the luminal surface of the choroid plexus and directly interact with CSF, thereby enabling more rapid interface immune responses (Cui et al., 2021; Lun et al., 2015; Xu et al., 2024).

**Figure 2.**
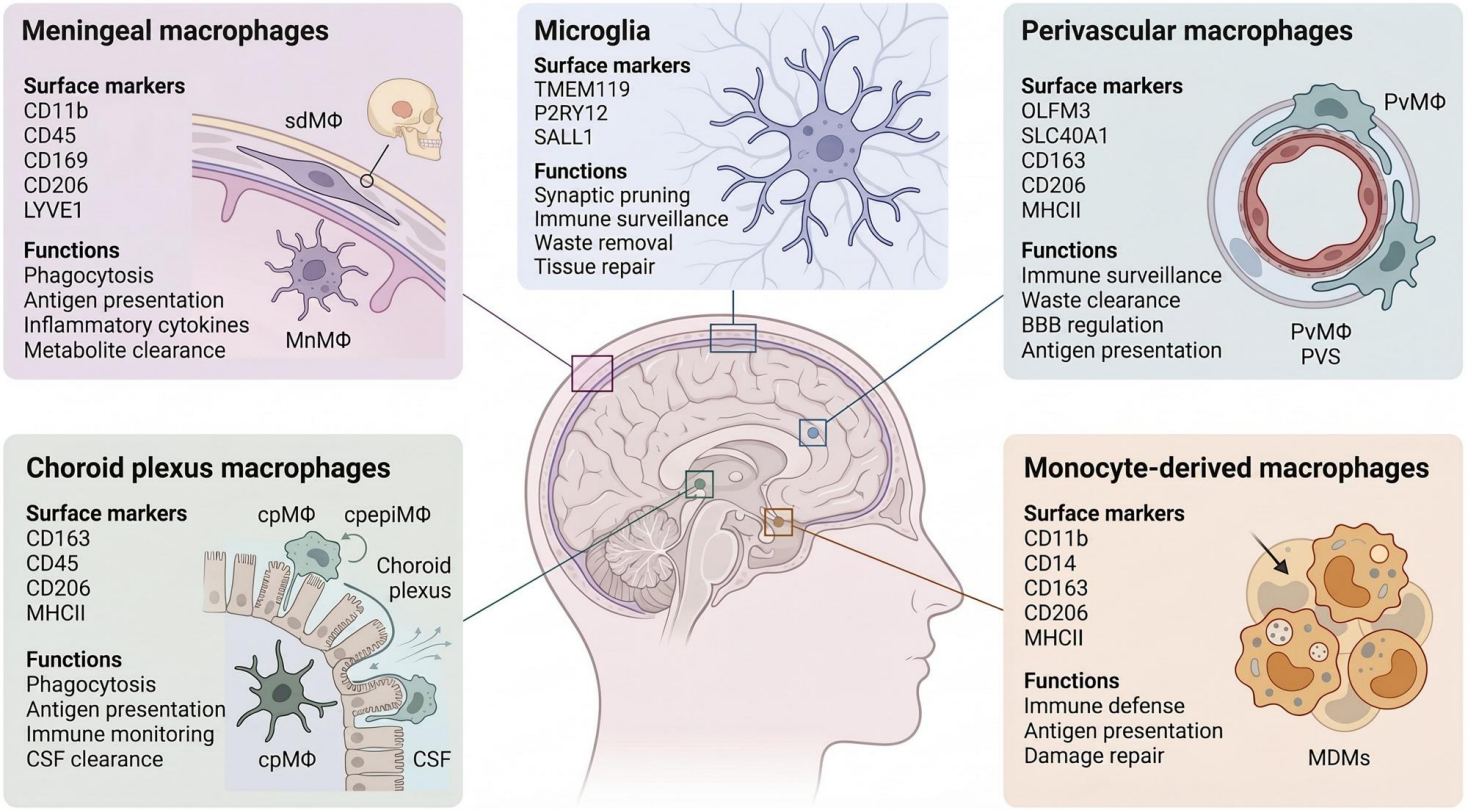
Spatial localization and interface-associated ecological niches of BAM subsets in the CNS, including sdMΦ/MnMΦ in the meninges, PvMΦ in perivascular spaces, and cpMΦ/cpepiMΦ in the choroid plexus.

Overall, the three major interfaces—the meninges, perivascular spaces, and choroid plexus—exhibit systematic differences in barrier permeability, geometric constraints, degree of exposure to humoral components, and sources of peripheral signaling input, and these factors collectively shape the morphological baseline, migratory behavior, and stress-responsive plasticity of distinct BAM subsets (Figure 2). This resulting “morphology-niche matching” framework not only accounts for the predominantly surveillance-oriented structural features of BAMs under homeostatic conditions but also provides the morphological substrate that enables their rapid transition to migratory and phagocytic response states upon microenvironmental perturbation, thereby establishing a spatial biological foundation for subsequent discussion of how tri-interface localization drives functional diversification.

Recent anatomical discoveries further expand this niche-based view of CNS borders. ACE points formed around bridging veins provide direct connections between the subarachnoid space and dura, creating potential routes for CSF-derived molecules and immune cells to communicate with dural immune compartments (Castellani et al., 2024; Smyth et al., 2024). DALT represents organized immune-cell aggregates along dural venous and lymphatic structures and may support local antigen sampling and humoral immune responses at the brain border (Park et al., 2024; Patel et al., 2025). The SLYM, a mesothelial membrane that subdivides the subarachnoid space, has been proposed to regulate solute transport, compartmentalize CSF flow, and provide a surface for immune surveillance near pial-perivascular routes (Møllgård et al., 2023). In addition, skull-dural and skull-meningeal channels connect skull bone marrow with the meninges and provide anatomical routes for myeloid-cell trafficking and inflammatory crosstalk between calvarial marrow and CNS borders (Mazzitelli et al., 2023; Pulous et al., 2022). These structures do not define new BAM subsets by themselves, but they refine the anatomical map within which BAMs sense antigens, sample CSF-derived signals, and interact with skull- or dura-derived immune inputs.

### Spatial Localization Shapes Functional Specialization

The three major interfaces—the meninges, perivascular spaces, and choroid plexus—exhibit systematic differences in anatomical position, barrier permeability, profiles of humoral exposure, and modes of peripheral signal input, and these differences primarily determine the distinct “information streams” encountered by BAMs. The meningeal compartment is more readily exposed to immune signals derived from skull bone marrow and the peripheral circulation and therefore tends to support sentinel functions such as immune surveillance and antigen presentation (Cugurra et al., 2021). The perivascular space, confined between two basement membranes and closely apposed to the neurovascular unit, is inherently positioned at the frontline of barrier stress and vascular wall-derived signaling, rendering it well suited for BBB monitoring, local inflammatory gating, and vascular-associated immune regulation (Hannocks et al., 2018; Ineichen et al., 2022). In contrast, the choroid plexus occupies the interface between blood and CSF, is directly linked to CSF composition and volume homeostasis, and consequently supports macrophage functions that emphasize continuous sensing of humoral fluctuations, rapid responsiveness, and maintenance of interface equilibrium (Cui et al., 2021; Dani et al., 2021; MacAulay et al., 2022). In this sense, the ecological niches of the three interfaces define distinct “priority surveillance targets” for BAMs—namely, peripheral immune inputs, vascular and barrier integrity, and CSF interface homeostasis—thereby providing a spatial biological starting point for subsequent discussion of functional specialization among BAM subsets.

Positional differences are further translated into executable cellular behavior programs through the interplay between spatial geometric constraints and morphological plasticity. The relatively confined meningeal space, in proximity to CSF pathways, biases MnMΦ toward a multiprocess, patrol-oriented surveillance morphology and enables their rapid transition to capture and phagocytosis under inflammatory conditions (Cui et al., 2021; Xu et al., 2024). The structural features of PvMΦ—namely, their longitudinal alignment and circumferential attachment along blood vessels—allow continuous, fine-scale sensing of vascular wall status and facilitate localized responses to barrier alterations (He et al., 2016; Wen et al., 2024). Within the choroid plexus, a complementary division of labor between relatively stationary stromal macrophages and luminally patrolling epithelial macrophages ensures sustained interface surveillance while permitting rapid redeployment to sites requiring immediate response (Cui et al., 2021; Xu et al., 2024). Thus, morphology is not an isolated phenotype but a niche-shaped functional “toolkit”: surveillance-associated processes enhance sampling and contact efficiency, whereas ameboid configurations optimize migration and phagocytosis, with transitions between these states occurring at distinct baselines and thresholds across the three interfaces.

From a functional perspective, BAMs across the three interfaces can be conceptualized as a spatial niche-driven continuum, ranging from immune sentinel and antigen-presenting roles in the meninges, through barrier gating and neurovascular unit regulation in the perivascular spaces, to maintenance of blood-CSF interface homeostasis and humoral immune control in the choroid plexus (Dalmau Gasull et al., 2024; Sun & Jiang, 2024). This logic—that positional differences dictate functional emphasis—also explains why BAMs frequently exhibit double-edged effects in disease contexts: the same immune surveillance and recruitment capacities may be beneficial during early infection or injury yet exacerbate immune entry and tissue damage under conditions of barrier vulnerability or chronic inflammation, while phagocytic and clearance functions can promote homeostatic recovery at specific stages but may also sustain local inflammatory circuits under persistent stimulation. To facilitate cross-compartmental comparison of BAMs with respect to ontogenetic origin, spatial localization, adult maintenance characteristics, marker combinations, and homeostatic functional bias, the relevant features are summarized in Table 1 (Cui et al., 2021; Vara-Pérez & Movahedi, 2025; Wen et al., 2024). Within this framework, subsequent sections addressing marker genes and transcriptional heterogeneity, regulatory networks, homeostatic functions, and disease mechanisms can be interpreted coherently as outcomes of how distinct interface niches shape cellular identity and state-transition thresholds, thereby ultimately determining BAM functional outputs and pathological consequences.

**Table 1.** Comparison of three BAM subsets.

BAM subset	Predominant origin	Typical location	Adult maintenance / turnover	Representative markers	Core functions
Meningeal macrophages (sdMΦ/MnMΦ) (Vara-Pérez & Movahedi, 2025)	Mainly yolk-sac EMPs; partial skull/peripheral bone-marrow input	Dura mater and leptomeninges	Mostly self-maintained; dural subsets more prone to peripheral replenishment after injury/inflammation	CD11b, CD45, CD206/LYVE1, MHC II	Interface immune surveillance, antigen presentation, CSF/meningeal homeostasis
Perivascular macrophages (PvMΦ) (Wen et al., 2024)	Predominantly embryonic EMPs; peripheral input in pathology	Perivascular spaces apposed to vessel walls between basement membranes	Low replacement at steady state; mixed origins can emerge with neuroinflammation/barrier disruption	CD11b, CD45, CD163, CD206, MHC II	BBB monitoring, neurovascular immune gating, context-dependent antigen presentation
Choroid plexus macrophages (cpMΦ/cpepiMΦ) (Cui et al., 2021)	Embryonic EMP-seeded; increasing monocyte contribution with age/perturbation	Choroid plexus stroma and epithelial/luminal surface, directly exposed to CSF	Mainly locally maintained; peripheral replenishment enhanced in permeable niches after perturbation	CD11b, CD45, CD163, CD206, MHC II	CSF-interface immune monitoring, barrier homeostasis, waste clearance, rapid immune responses

Note: EMPs, erythro-myeloid progenitors; PVS, perivascular space; BBB, blood-brain barrier; CSF, cerebrospinal fluid. Marker usage varies across studies; panels should be interpreted together with anatomical localization and experimental context to minimize subset misclassification.

## Marker Genes and Transcriptomic Characteristics of BAMs

BAMs express macrophage-lineage markers together with compartment-enriched markers that help distinguish them from microglia and infiltrating monocyte-derived macrophages. Advances in scRNA-seq, high-resolution imaging, gene-editing approaches, and spatial transcriptomics have refined BAM identification across the meninges, choroid plexus, and perivascular spaces (Brioschi et al., 2023; Masuda et al., 2022). However, because individual markers can overlap across subsets, activation states, species, and disease contexts, BAM classification requires the combined interpretation of anatomical location, multi-marker panels, and transcriptional modules rather than reliance on a single marker (Dalmau Gasull et al., 2024; Penati et al., 2025).

### Combinatorial Marker Systems Across BAM Subsets

BAM marker architecture can be viewed as a hierarchy consisting of a shared macrophage core and compartment-specific marker combinations. Common myeloid/macrophage markers include CD45, CD11b, CD64, F4/80, MerTK, and CX3CR1, whereas antigen-presentation-associated states are often reflected by MHC II and CD11c expression (Dermitzakis et al., 2023; Rebejac et al., 2022; Sun & Jiang, 2024). CD206/MRC1, LYVE1, CD163, CD169, and MHC II are frequently used to support BAM identification, but none is sufficiently specific alone; therefore, their interpretation should be anchored to tissue location and exclusion of microglial markers such as TMEM119, P2RY12, and SALL1.

At the subset level, CD11c is relatively enriched in cpepiMΦ and is associated with antigen presentation and immune surveillance, whereas CD206/MRC1 is commonly used for PvMΦ and choroid plexus macrophages and is linked to phagocytosis and immune regulation (Cui et al., 2021; Masuda et al., 2022). Because CD206^+^ and LYVE1^+^ definitions are not fully interchangeable across studies, a robust strategy is to combine spatial localization with marker panels including CD163, CD206, LYVE1, CD169, MHC II, and CD11c (Utz et al., 2020).

Compartmental analyses show that MnMΦ, PvMΦ, cpMΦ, and cpepiMΦ display overlapping but distinguishable marker patterns, with additional species differences between human and mouse datasets (Sun & Jiang, 2024; Utz et al., 2020; Vara-Pérez & Movahedi, 2025). For this reason, Table 2 summarizes recommended marker combinations and use-cases for distinguishing BAMs from microglia and MDMs, while emphasizing that subset assignment should be validated by anatomical context whenever possible (Brioschi et al., 2023; Kenkhuis et al., 2022; Mrdjen et al., 2018; Rebejac et al., 2022; Robert et al., 2023; Sun & Jiang, 2024; Vara-Pérez & Movahedi, 2025).

**Table 2.** Markers and use-cases to distinguish BAMs, microglia, and monocyte-derived macrophages.

Use-case	Recommended marker panel	Expected readout for BAMs	Expected readout for microglia	Expected readout for MDMs
Baseline CNS myeloid calling (Mrdjen et al., 2018)	CD45, CD11b, CD64, MerTK	Macrophage-lineage; proceed to BAM panels	Often CD45 low myeloid; proceed to microglia markers	Usually rare at steady state; suggests pathology if present
Most common confusion: BAMs vs microglia (Brioschi et al., 2023)	CD163/CD206/LYVE1 + TMEM119/P2RY12/SALL1	CD163/CD206/LYVE1 enriched	TMEM119/P2RY12/SALL1 enriched	Partial overlap possible; require infiltration context
Sub-compartment hinting with known anatomy (Sun & Jiang, 2024)	Mouse: CD206, CD169; Human: CD163, CD14, LYVE1 (with location)	Distinct patterns across meninges/PVS/CP	Not primary for assignment	Not for compartment assignment
Antigen-presentation-leaning states (Rebejac et al., 2022)	MHC II low/high + CD11c	Subset with MHC II high and/or CD11c high	MHC II can rise in subsets; not standalone	Often high in inflammation; needs context
Inflammation/infection state mapping (Robert et al., 2023)	IL1B, IL6, TNF, CXCL10 (±TLR4, NOS2)	Inflammatory upshift with state change	Similar upshift; confirm identity markers	Typically strongest inflammatory program; highest confusion risk
AD-like state cue (Vara-Pérez & Movahedi, 2025)	APOE, TREM2 + identity panel	APOE/TREM2 up in boundary states	Similar induced states possible	Can express in some models; check infiltration
Do-not-use-alone markers (Kenkhuis et al., 2022)	P2RY12 alone; CD206 alone; LYVE1 alone; MHC II alone	Single markers misclassify subset/state	P2RY12 outside microglia needs caution	Activation can mimic; single markers unreliable

### Subset States and Transcriptional Dynamics

The transcriptomic characteristics of BAMs reveal substantial functional diversity and plasticity under both homeostatic and pathological conditions. Under steady-state conditions, BAMs at distinct interfaces display gene expression patterns aligned with their niche-specific tasks; for example, choroid plexus BAMs express genes associated with CSF barrier function, such as Ttr, whereas perivascular BAMs preferentially express genes related to vascular barrier maintenance, including Lyve1 (Wen et al., 2024; Xu et al., 2024). In meningeal BAMs, the selective expression of Clec4n, Clec10a, and Folr2 is potentially linked to antigen-presenting functions (Schonhoff et al., 2023). At the same time, genes such as Cybb, Apoe, Ms4a7, Tgfb1, and Mrc1 (CD206) are highly expressed across multiple BAM subsets, indicating a shared core transcriptional module associated with lipid metabolism and immunoregulatory processes (Jordão et al., 2019; Sun & Jiang, 2024). This organization—comprising a conserved core program alongside compartment-specific modules—provides a molecular basis for understanding how BAMs support distinct homeostatic functions across brain interfaces.

Under pathological conditions, the transcriptomic profiles of BAMs undergo marked alterations, characterized by the upregulation of gene programs associated with inflammatory responses, immune regulation, and cellular recruitment. During neuroinflammation, BAMs markedly increase expression of proinflammatory cytokines, including IL-1β, IL-6, and TNF-α, as well as chemokines such as CXCL10 and CX3CL1, which play central roles in orchestrating inflammatory cascades and immune cell recruitment (Schonhoff et al., 2023; Xu et al., 2024). Following infection, BAMs upregulate immune response-related genes and proinflammatory mediators while concomitantly downregulating homeostasis-associated genes, suggesting the occurrence of transcriptional reprogramming; however, these changes vary across cellular subsets and stimulus contexts and therefore require finer-grained stratification and spatiotemporal analyses for resolution (Da Mesquita & Rua, 2024; Robert et al., 2023). In addition, during neuroinflammatory states, BAMs may increase expression of genes linked to oxidative stress and phagocytic activity, which, under certain conditions, can exacerbate inflammatory responses and tissue damage (Mendiola et al., 2022).

**Figure 3.**
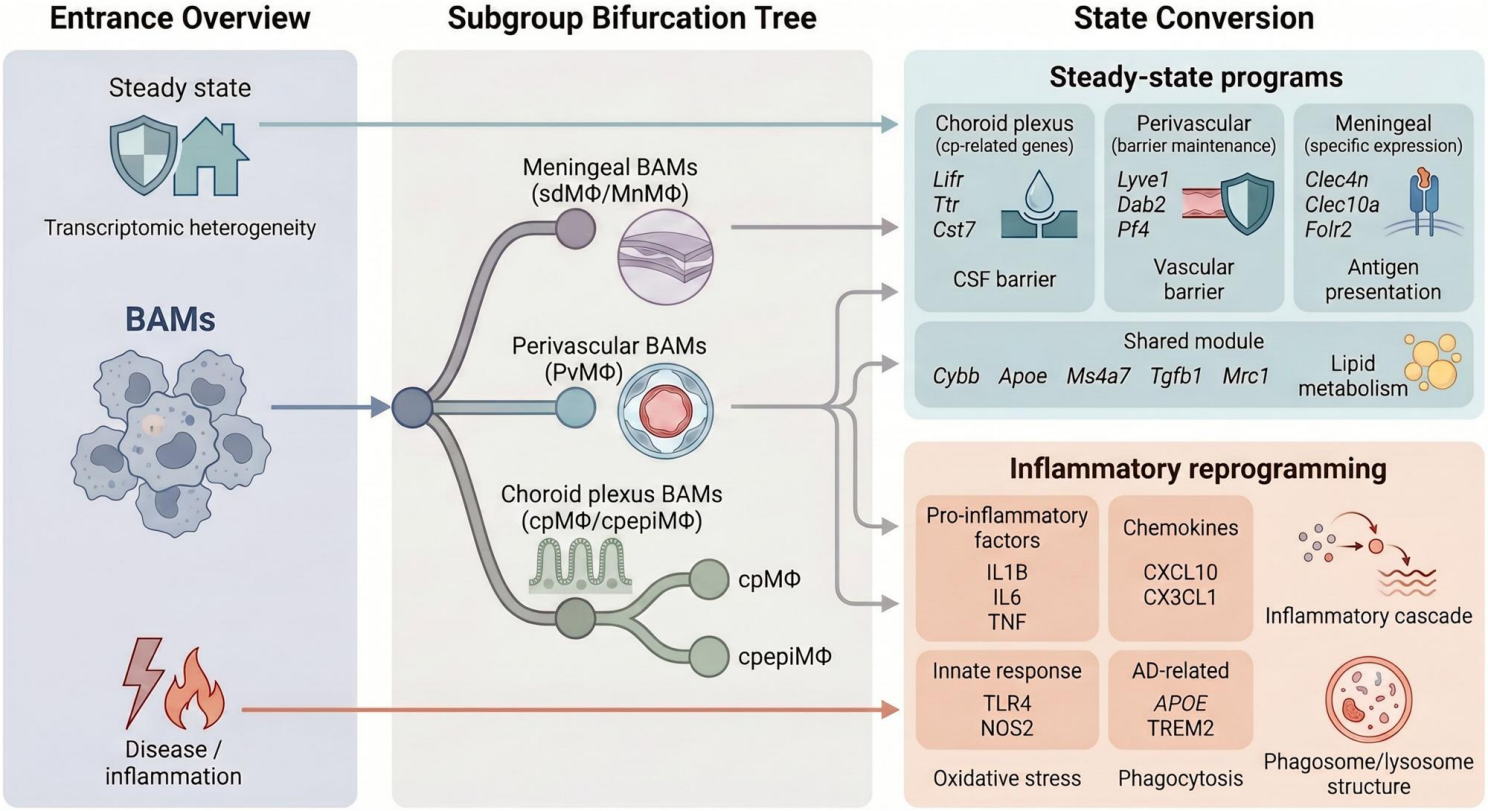
Conceptual framework illustrating the transcriptional heterogeneity of BAM subsets, including MnMΦ, PvMΦ, cpMΦ, and cpepiMΦ, and their distinction from microglia and MDMs.

Transcriptional alterations in BAMs are also observed across multiple disease contexts. In AD, BAMs upregulate genes associated with neuroinflammation and amyloid deposition, such as Apoe and Trem2 (Vara-Pérez & Movahedi, 2025). Disruption of CX3CR1 signaling may exacerbate neuronal injury by modulating the release of inflammatory mediators, a mechanism that has been validated in copper-induced demyelination models (Mendiola et al., 2022). At the same time, the transcriptomic features of BAMs exhibit dynamic changes during development: embryonic BAM precursors express genes involved in cellular differentiation and migration, including CX3CR1 and F4/80, indicating that their identity is shaped by lineage-specific programs from early developmental stages (Kierdorf et al., 2013; Masuda et al., 2022; Utz et al., 2020). Taken together, these findings indicate that BAMs display compartment-specific functional transcriptional programs under homeostatic conditions and undergo substantial transcriptional reprogramming in response to inflammatory or disease-associated stimuli (Figure 3).

### Transcriptional Boundaries Among Adjacent Myeloid Populations

Within the CNS immune cell landscape, BAMs frequently exhibit phenotypic overlap with microglia and with MDMs that infiltrate under pathological conditions, making the establishment of clear transcriptional boundaries particularly critical for accurate functional attribution (Da Mesquita & Rua, 2024; Munro et al., 2022). Current RNA sequencing evidence indicates that rat CD163^+^ BAMs display transcriptional signatures characterized by genes such as Clec10a, Apoc1, and Cxcr4, whereas microglia are typified by markers including Tmem119 and P2ry12, which show low expression in BAMs (Jurga et al., 2020; Penati et al., 2025; Rajan et al., 2020). It should be noted that P2ry12 is widely regarded as a canonical microglial marker, yet its expression and regulation in BAMs require more specific validation, underscoring that cell classification should not rely on single-gene criteria alone (Jurga et al., 2020; Penati et al., 2025). In addition, stratification of BAMs based on MHC class II expression (MHC II^low^ versus MHC II^high^) correlates with antigen-presenting potential and provides an additional dimension for distinguishing immune functional states (Da Mesquita & Rua, 2024; Rebejac et al., 2022; Schonhoff et al., 2023).

Conversely, during neuroinflammation, infection, or other pathological processes, BAMs undergo inflammation-associated transcriptional reprogramming and upregulate a range of proinflammatory cytokines, chemokines, and immune response genes; concurrently, peripherally infiltrating monocytes/MDMs carry similarly robust inflammatory and recruitment-related transcriptional programs, and partial overlap at the transcriptomic level further complicates cellular identity assignment (Munro et al., 2022; Rajan et al., 2020). Accordingly, a more robust discrimination strategy integrates spatial localization (meningeal, perivascular, or choroid plexus interfaces), combinatorial marker systems (e.g., CD163, CD206, LYVE1, CD169, MHC II, and CD11c), and transcriptional module weighting (the relative contribution of homeostatic maintenance versus inflammatory response programs) to minimize misclassification of infiltrating cells as resident BAMs, or vice versa. Importantly, recent single-cell studies indicate that human BAM populations are more complex than those in mice, underscoring the need for cautious cross-species comparisons and continued refinement of identification frameworks for human data interpretation (Zhan et al., 2025).

Overall, BAMs possess a shared macrophage marker core that supports identity recognition, while forming compartment-specific marker combinations and transcriptional programs across distinct interfaces, including the meninges, perivascular spaces, and choroid plexus; scRNA-seq and spatial transcriptomic approaches have further confirmed their transcriptional heterogeneity and state plasticity and have identified discernible functional modules under both homeostatic and pathological conditions (Sun & Jiang, 2024; Vara-Pérez & Movahedi, 2025). However, heterogeneity in definitional criteria across studies, reliance on single-marker classification schemes (e.g., CD206^+^ versus LYVE1^+^), incomplete correspondence of marker systems between humans and mice, transcriptional overlap introduced by inflammatory reprogramming and infiltrating cells under pathological conditions, and unresolved specificity of canonical microglial markers (such as P2ry12) within BAMs remain major sources of inconsistency across reported findings (Dalmau Gasull et al., 2024; McKinsey et al., 2020; Sun & Jiang, 2024). Therefore, in subsequent discussions of regulatory networks, homeostatic functions, and disease-associated double-edged effects, it is necessary to adopt a unified interpretative framework integrating spatial localization, combinatorial marker usage, and transcriptional module weighting to reconcile divergent observations and enhance cross-study comparability.

## Regulatory Networks of BAMs

### Core Transcription Factor Modules for Cell Identity Maintenance

The development, maintenance, and functional execution of BAMs are tightly regulated by multiple signaling molecules and transcription factors (Brioschi et al., 2025). Among these, PU.1 and IRF8 constitute the core transcriptional scaffold of myeloid and interface macrophages, whereas the requirement for MAFB varies across different CNS-resident macrophage populations (Penati et al., 2025; Yamasaki et al., 2024). PU.1 (SPI1) serves as a master regulator of myeloid differentiation and forms the foundational layer of the regulatory network underlying macrophage subset formation (Dermitzakis et al., 2023). Its expression level critically influences precursor cell fate decisions: high PU.1 expression preferentially promotes macrophage differentiation, whereas lower PU.1 levels bias differentiation toward the granulocytic lineage (DeKoter et al., 2007). More importantly, sustained PU.1 activity regulates the expression of CSF1R, TLRs, CX3CR1, and molecules involved in adhesion and migration, thereby endowing BAMs with phagocytic capacity, environmental sensing, and tissue-localization competence; its regulation of A1/A2 progenitor populations is indispensable, as genetic ablation results in embryonic lethality, underscoring its essential role in early development (Kierdorf et al., 2013; Lian et al., 2023; McKercher et al., 1996).

Building upon this foundation, IRF8 further specifies and maintains BAM identity and participates in the activation of immune-related transcriptional programs (Yamasaki et al., 2024). PU.1 and IRF8 act cooperatively to establish a fully functional population of BAMs (Kierdorf et al., 2013). Loss of IRF8 disrupts the core transcriptional programs of microglia and CNS interface macrophages, largely because precursor cells fail to differentiate into mature phenotypes with BAM characteristics (Yamasaki et al., 2024). Within BAMs, IRF8 contributes to the maintenance of core transcriptional identity and correlates with gene programs involved in antigen presentation, phagocytic clearance, and interface immune surveillance, thereby potentially influencing immune homeostasis and barrier-associated microenvironmental stability at brain borders (Hagemeyer et al., 2016; Yamasaki et al., 2024).

MAFB plays a more prominent role in the terminal differentiation and homeostatic functional specialization of macrophages, particularly in suppressing inflammation and sustaining anti-inflammatory phenotypes and metabolic adaptation. Its dependency varies among CNS-resident macrophage populations, being more pronounced in microglia and comparatively weaker in BAMs (Penati et al., 2025; Yamasaki et al., 2024). MAFB influences cellular anchoring and morphology by targeting genes such as Cx3cr1, S1pr1, and Cd209a, and promotes an anti-inflammatory orientation through suppression of NF-κB-associated proinflammatory programs (Cuevas et al., 2017; Kim, 2017). More broadly, MAFB appears to support long-term tissue residency and pro-resolving functions by regulating the coupling between macrophage metabolism and inflammation, for example, by facilitating PGE2-mediated lipid mediator class switching via the COX-2/PGE2/EP4-ALOX15 axis and by constraining NLRP3 inflammasome activation through maintenance of mitochondrial homeostasis (Cui et al., 2023; Hamada et al., 2020; Kanai et al., 2024).

### Survival Signaling Axes Governing Developmental Seeding and Resident Maintenance

Complementing the “identity maintenance module,” a set of survival signaling axes determines BAM abundance, viability, and homeostatic renewal capacity. Central to this regulation is the CSF-1/IL-34-CSF1R pathway and its regulatory element FIRE, with upstream developmental and epigenetic locking provided by Runx1 (Rojo et al., 2019; Van Hove et al., 2025). Colony-stimulating factor-1 (CSF-1, also known as M-CSF), produced by stromal and epithelial cells of the choroid plexus, functions as a key factor throughout the macrophage life cycle by regulating lineage commitment, differentiation, survival, proliferation, and effector function (Hamilton & Achuthan, 2013; Jones & Ricardo, 2013). Macrophages express high levels of CSF1R, and binding of CSF-1 or IL-34 activates downstream signaling pathways, including PI3K/Akt, MAPK, and JAK/STAT, thereby promoting survival and self-renewal; in the absence of CSF-1, macrophages rapidly undergo apoptosis (Busca et al., 2014; Sehgal et al., 2021; Sinha et al., 2021). Within BAM populations, CSF-1 preferentially supports early generation, whereas IL-34 is particularly critical for adult homeostatic maintenance; IL-34 deficiency results in reduced BAM numbers accompanied by aberrant expression of identity markers such as MHC II, CD206, and CD163 (Van Hove et al., 2025).

FIRE, located within the first intron of the Csf1r gene, is considered a key regulatory switch that determines macrophage lineage identity. By recruiting PU.1 and establishing an open chromatin configuration, FIRE enhances Csf1r promoter activity, thereby increasing progenitor responsiveness to M-CSF and sustaining macrophage development and homeostasis (Sauter et al., 2013). In FIRE-deficient mutant mice, choroid plexus macrophage numbers are markedly reduced and accompanied by functional impairments (Rojo et al., 2019). Other studies have reported that FIRE-deficient mice lack microglia while retaining BAMs, suggesting differential FIRE dependency among brain macrophage populations and offering insight into compartment-specific differences in BAM homeostatic stability (Munro et al., 2020).

Runx1 operates further upstream by shaping developmental programs and establishing epigenetic imprints required for tissue residency. Development of YS-derived macrophages, including BAM precursors, depends on Runx1 but not on hematopoietic stem cells, as Runx1 drives progenitor commitment toward macrophage lineages while suppressing granulocytic and erythroid differentiation (Tober et al., 2013; Utz et al., 2020). Loss of Runx1 results in the absence of BAMs in the choroid plexus and meninges, accompanied by mislocalization, underscoring its essential role in CNS border seeding and stable residency (Tober et al., 2013). As a central scaffold of the myeloid transcriptional network, PU.1 cooperates with environment-induced secondary transcription factors to select and activate tissue-specific enhancers, thereby fixing the phenotypic identity and functional thresholds of tissue-resident macrophages (Gosselin et al., 2014).

### Communication Receptor Modules for Interface Sensing and Homeostatic Gating

At CNS borders, BAMs must translate signals derived from the vascular wall, CSF, and neighboring cells into surveillance-response thresholds, and the CX3CR1-CX3CL1 axis constitutes a key neuroimmune communication module, with particularly prominent expression in choroid plexus macrophages and PvMΦ (Cui et al., 2021; Pawelec et al., 2020; Wen et al., 2024;). In developmental and homeostatic studies, CX3CR1 has been widely used as a tracer and classification marker for interface-associated myeloid cells (Utz et al., 2020). At the level of spatial organization, PvMΦ and choroid plexus-associated macrophages are often distributed along their respective interface structures and exhibit morphologies consistent with interface surveillance; these spatial features provide the structural basis for immune surveillance and localized regulation at vascular and humoral interfaces (Cui et al., 2021; Wen et al., 2024). In microglia, CX3CL1-CX3CR1 signaling has also been shown to constrain excessive inflammatory responses by activating NRF2-associated antioxidant pathways (Castro-Sánchez et al., 2019). Accordingly, based on the current body of evidence, a more precise interpretation is that the CX3CL1-CX3CR1 axis provides CNS border myeloid cells with an essential neuroimmune communication gateway, whereas long-term maintenance of BAMs and compartmental homeostasis is primarily supported by survival and renewal pathways such as CSF1R signaling; together, these systems shape BAM state transitions between homeostatic surveillance and stimulus-responsive activation (Goldmann et al., 2016; Sun & Jiang, 2024; Van Hove et al., 2025).

In addition to CX3CR1-mediated communication, TLR4 should be considered an important context-dependent regulatory receptor for BAM state transitions. At the choroid plexus, meningeal, and perivascular interfaces, TLR4 enables BAMs to sense pathogen-associated and damage-associated molecular patterns, including lipopolysaccharide and ischemia-related DAMP signals, and to engage MyD88/TRIF-linked NF-κB and interferon-associated programs that promote cytokine and chemokine production (Beuker et al., 2022; Robert et al., 2023; Yu et al., 2026). Because TLR4 is broadly expressed across myeloid populations and can increase during inflammation, it should not be used as a lineage-defining marker; rather, it is best interpreted as a regulatory axis that modulates inflammatory responsiveness, antigen-processing tone, and recruitment signals within anatomically defined BAM niches.

### An Integrated Framework for State Transitions and Disease-Associated Reprogramming

Overall, state transitions in BAMs can be understood as the outcome of coordinated multilayered regulatory networks: myeloid core transcription factors such as PU.1 and IRF8 maintain a permissive “transcriptional chassis,” survival and homeostatic renewal are sustained by CSF1R and its ligands (CSF-1 and IL-34) together with regulatory elements such as Csf1r-FIRE, and niche-associated receptor axes, including CX3CL1-CX3CR1 and TLR4-dependent danger-sensing pathways, contribute to interface signal integration and response-threshold modulation (Brioschi et al., 2025;; Munro et al., 2020; Yamasaki et al., 2024; Van Hove et al., 2025). Within this framework, PU.1 provides the foundational transcriptional infrastructure for macrophage receptor expression and phagocytic programs; IRF8 contributes to maintaining the core transcriptional identity of CNS interface macrophages and links this identity to immune-related programs; MAFB tunes anti-inflammatory and metabolic states; and TLR4 amplifies stimulus-induced inflammatory outputs when BAMs encounter microbial or damage-associated signals. In parallel, the CSF-1/IL-34-CSF1R axis and regulatory elements such as FIRE provide sustained survival and renewal licensing, while Runx1 acts primarily at upstream developmental stages to influence the establishment of tissue-resident macrophage pools (Buchrieser et al., 2017; Mass et al., 2016; Van Hove et al., 2025; Yamasaki et al., 2024) (Table 3) (Amann et al., 2023; Barrachina et al., 2022; Beuker et al., 2022; Brioschi et al., 2020; Buchrieser et al., 2017; Castro-Sánchez et al., 2019; Dermitzakis et al., 2023; Gerlach et al., 2016; Ginhoux et al., 2010; Goldmann et al., 2016; Gosselin et al., 2014; Hagemeyer et al., 2016; Hamada et al., 2020; Hamilton & Achuthan, 2013; Kelly et al., 2000; Kierdorf et al., 2013; Kim et al., 2021; Koshida et al., 2017; Lee et al., 2021; Mass et al., 2016; Munro et al., 2020; Owens et al., 2022; Robert et al., 2023; Rojo et al., 2019; Sankowski et al., 2021; Sauter et al., 2013; Sehgal et al., 2021; Siret et al., 2022; Utz et al., 2020; Van Hove et al., 2019; 2025; Yadav et al., 2020; Yamasaki et al., 2024; Yu et al., 2026).

**Table 3.** Regulatory factors in BAMs: downstream effects and evidence types.

Regulatory factor	Major pathways / targets	Stage of action	Key evidence type and takeaway
PU.1 (SPI1) (Goldmann et al., 2016)	CSF1R, TLRs, CX3CR1; adhesion/migration programs	Early differentiation; adult identity maintenance	KO embryonic lethality; regulates A1/A2 precursors and myeloid fate; supports receptor/positioning programs (Buchrieser et al., 2017; Dermitzakis et al., 2023)
IRF8 (Yamasaki et al., 2024)	Cooperates with PU.1; CD206/TIM4/LYVE1; MARCO, CX3CR1	Differentiation & identity; immune program activation	Irf8 KO: meningeal/CP BAMs largely absent while microglia remain; indicates failed differentiation (Kierdorf et al., 2013; Van Hove et al., 2019; Yamasaki et al., 2024)
CSF-1 (M-CSF) (Van Hove et al., 2025)	CSF1R→PI3K/Akt, MAPK, JAK/STAT	Lifelong survival/proliferation	CSF-1 deprivation triggers rapid apoptosis; supports BAM generation/maintenance (Ginhoux et al., 2010; Hamilton & Achuthan, 2013; Munro et al., 2020; Sehgal et al., 2021)
IL-34 (Van Hove et al., 2025)	IL-34-CSF1R homeostasis; affects MHC II/CD206/CD163	Adult steady-state maintenance	IL-34 deficiency reduces BAM numbers (notably PVMs) and alters identity markers (Van Hove et al., 2025)
FIRE (Rojo et al., 2019)	Csf1r intronic enhancer; PU.1 recruitment ↑CSF1R	Receptor-expression licensing; homeostasis	FIRE loss reduces cpMΦ with functional deficits; dependence may differ by subset (Koshida et al., 2017; Rojo et al., 2019; Sauter et al., 2013)
MAFB (Yamasaki et al., 2024)	NF-κB suppression; PGC-1α/mitochondria; Claudin-5/Occludin/Timp1	Terminal differentiation; anti-inflammatory/metabolic tuning	Upregulated from late embryogenesis; suppresses pro-inflammatory cytokines; supports barrier/structure integrity (Amann et al., 2023; Hamada et al., 2020; Kelly et al., 2000; Koshida et al., 2017; Yadav et al., 2020; Yamasaki et al., 2024)
Runx1 (Brioschi et al., 2020)	Yolk-sac commitment; enhancer cooperation (PU.1/IRF8); Cx3cr1, Itga4/Itgb1; represses Nos2/IL-12b; supports MAFB/IL-10	Embryonic seeding; residency maintenance	Runx1 loss abolishes meningeal/CP BAMs and disrupts positioning; supports epigenetic/anchoring programs (Goldmann et al., 2016; Gosselin et al., 2014; Lee et al., 2021; Mass et al., 2016; Owens et al., 2022; Utz et al., 2020; Van Hove et al., 2025)
CX3CR1 (Siret et al., 2022)	CX3CL1-guided seeding; restricts egress; TLR4 and MHC II trafficking; morphology/mesh	Seeding; surveillance gating; response thresholding	Developmental and adult residency evidence; loss increases apoptosis; promotes LPS responsiveness and antigen presentation (Barrachina et al., 2022; Castro-Sánchez et al., 2019; Gerlach et al., 2016; Hagemeyer et al., 2016; Kim et al., 2021; Sankowski et al., 2021)
TLR4	DAMP/PAMP sensing; MyD88/TRIF-NF-κB/IRF3	Stimulus-induced inflammatory activation	Promotes cytokine/chemokine output during infection and ischemic injury; not BAM-specific, so interpret with identity markers (Beuker et al., 2022; Robert et al., 2023; Yu et al., 2026)

**Figure 4.**
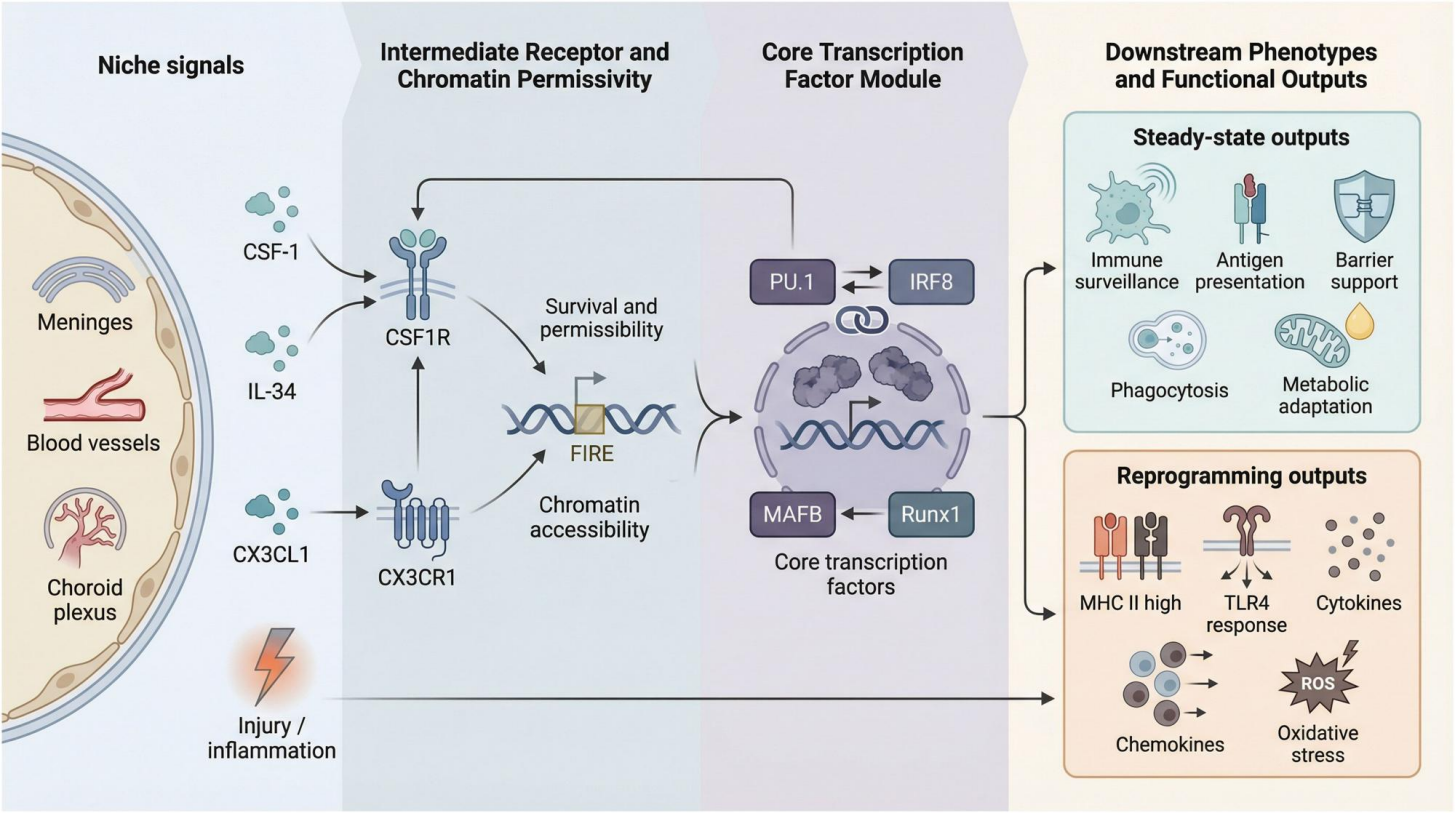
Schematic representation of the regulatory networks governing BAM subsets, including PU.1/IRF8/MAFB transcriptional modules, CSF1R-dependent survival signaling, CX3CR1 communication, and TLR4-mediated danger sensing.

This integrative perspective also facilitates the interpretation of the double-edged roles of BAMs in disease. In contexts such as infection or neuroinflammation, BAMs can amplify border immune responses through programs involving antigen presentation and immune cell recruitment, whereas under conditions of chronic inflammation or barrier vulnerability, these same responses may promote immune infiltration and secondary tissue damage. At the same time, enhanced recruitment and entry of myeloid cells into CNS border regions during pathological states further increase transcriptional state overlap and complicate functional attribution (Figure 4).

## Roles of BAMs in Brain Homeostasis

BAMs are lifelong residents of CNS border regions and function as immune sentinels, playing indispensable roles in maintaining brain homeostasis. As the first line of defense of the CNS, BAMs are chronically exposed to CSF flow and the microenvironments of boundary interfaces, where they perform long-range surveillance and sensing of the brain milieu through the secretion of diverse mediators, including cytokines and neurotrophic factors; in doing so, they support the normal function of neurons and glial cells and help preserve intracerebral homeostasis and equilibrium (Castellani et al., 2023; Silvin et al., 2023). When damage-induced chemotactic signals or pathogen-associated molecular patterns arise at boundary regions, BAMs rapidly transition into an activated state, initiate immune responses, and modulate local immune processes, thereby providing additional support to resident microglia in certain contexts and protecting the brain from infection and injury (Pedragosa et al., 2018; Rustenhoven & Kipnis, 2022; Xu et al., 2024). At the homeostatic level, BAM functions extend beyond singular immune defense to encompass barrier maintenance, metabolic waste clearance, and support of neuronal activity, and these multidimensional outputs are closely aligned with the ecological niches of the meninges, choroid plexus, and perivascular spaces in which they reside.

### Homeostatic Functions of MnMΦ

MnMΦ are positioned at the frontline of CNS borders and represent one of the earliest responders to microbial invasion of the brain; their homeostatic functions are therefore first manifested as continuous surveillance of pathogens and damage-associated signals, coupled with fine-tuned setting of inflammatory thresholds (Rustenhoven & Kipnis, 2022). They detect potential invasive cues through TLRs and scavenger receptors and can engage interferon responses and chemokine networks to recruit peripheral immune cells for host defense (Kim et al., 2023; Rebejac et al., 2022). At the same time, MnMΦ are not constitutively proinflammatory: under homeostatic conditions, they secrete TGF-β to maintain immune tolerance, whereas under pathological conditions, they can shift toward a proinflammatory phenotype (e.g., iNOS^+^ expression) and exacerbate neuroinflammation, highlighting pronounced state plasticity between defensive and tolerogenic programs (Ding et al., 2026). This “immune surveillance-threshold gating” framework enables MnMΦ to rapidly initiate boundary defense while minimizing the risk of excessive inflammation in the adjacent, vulnerable neural tissue.

MnMΦ also contributes to establishing a “controlled interface immune environment” through interactions with boundary vascular structures and meningeal immune pathways. On the one hand, regions such as the dural venous sinuses are regarded as critical neuroimmune interfaces, where MnMΦ cooperate with adjacent vascular and stromal cells to shape local immune niches, thereby supporting the ordered surveillance of antigens and danger signals under homeostatic conditions and influencing immune cell retention and migration at the meningeal boundary (Rustenhoven et al., 2021). It should be emphasized that tight junction molecules such as CLDN5 and OCLN are primarily components of the barrier phenotype of vascular or barrier-associated cells rather than direct molecular mechanisms by which MnMΦ maintain barrier integrity; instead, MnMΦ more likely exert indirect effects on boundary permeability and immune cell recruitment through their perivascular niche positioning and through networks of inflammatory mediators and chemokines (Rustenhoven et al., 2021; Weller et al., 2018). On the other hand, local immunoregulatory interactions that restrain excessive T cell activation are also present within the meninges: for example, mural cells in the dura mater can establish contact with macrophages and transfer cytoplasmic components, thereby suppressing antigen-dependent helper T cell activation and limiting TH17 differentiation, ultimately providing a finely tuned brake on autoimmune-like responses at the boundary (Min et al., 2024). Functional evidence further supports the critical role of MnMΦ in anti-infectious homeostatic defense: experimental perturbation or depletion of MnMΦ compromises control of lymphocytic choriomeningitis virus-associated neuroinfection in mice, leading to increased viral burden and more severe outcomes; moreover, MHC II^hi^ macrophages enriched around adult dural venous sinuses exhibit stronger antiviral and chemokine-related signatures and confer protection against systemic viral entry into the CNS (Kim et al., 2023; Rebejac et al., 2022). Notably, these protective effects are closely linked to interferon-associated antiviral programs, while the specific division of labor among MnMΦ subsets across distinct anatomical sites and phenotypic states, as well as the underlying mechanisms, remains to be further elucidated (Kim et al., 2023; Rebejac et al., 2022).

### Choroid Plexus Macrophages

Choroid plexus macrophages are positioned at the critical interface between the blood and CSF and act as key executors of CSF immune surveillance; their homeostatic functions are first reflected in the continuous monitoring and rapid clearance of pathogens and exogenous substances within the CSF (Cui et al., 2021; Xu et al., 2024). They recognize pathogen-associated molecular patterns, such as lipopolysaccharide, via TLR4 and phagocytose pathogens and foreign materials through receptors including CD206, thereby maintaining immune surveillance and cleanliness of the CSF environment (Cui et al., 2021; Robert et al., 2023). Choroid plexus BAMs can also secrete IFN-λ to block viral entry into the brain parenchyma via the CSF; in the context of HSV-1 infection, their phagocytic efficiency can even determine whether viruses breach the blood-CSF barrier, highlighting a critical “interception” role in interface antiviral defense (Lazear et al., 2015; Wilcox et al., 2016). Accordingly, choroid plexus macrophages are not merely passive resident cells but constitute continuously operating immune-sensing and clearance units at the CSF interface.

With respect to barrier integrity and substance exchange, choroid plexus macrophages help limit CSF leakage and maintain barrier stability by modulating epithelial tight junctions and interface permeability. Evidence indicates that, under inflammatory or injury conditions, choroid plexus macrophages engage in bidirectional interactions with epithelial cells and participate in interface repair and in regulating barrier function (Xu et al., 2024). In parallel, through phagocytosis and immune surveillance, choroid plexus macrophages facilitate the handling and regulation of antigens and metabolites and participate in CSF clearance and circulation: they engulf and remove intraventricular metabolic byproducts, cellular debris, and other waste materials, thereby preserving CSF cleanliness and supporting normal circulation and absorption (Cui et al., 2021). Moreover, the choroid plexus is considered a major checkpoint and signaling hub for peripheral immune cell entry into the CNS; following inflammatory activation, immune cells can access the CSF via distal choroid plexus villi, indicating that this interface serves a “selective gatekeeping” function under both homeostatic and stress conditions (Robert et al., 2023; Schwerk et al., 2015; Vara-Pérez & Movahedi, 2025).

From the perspective of homeostatic signaling axes, the functions of choroid plexus macrophages can be summarized as a triad of “immune surveillance-barrier reinforcement-CSF clearance.” TLR4/CD206-mediated recognition and phagocytosis provide continuous immune scanning; mediators such as ANG-1 and TGF-β enhance barrier integrity by reinforcing epithelial tight junctions; and the phagocytic removal of metabolic waste and cellular debris supports CSF circulation and humoral homeostasis. In addition, choroid plexus BAMs exert regulatory effects on epithelial cell function: by influencing epithelial channel and transport processes, dysregulation of these macrophages can induce excessive CSF production and has been linked to hydrocephalus, indicating that their homeostatic roles extend beyond immune defense to encompass regulation of CSF production and fluid balance (Robert et al., 2023; Wang et al., 2024).

### PvMΦ

PvMΦ occupy a strategic position within the neurovascular unit, and their homeostatic functions are primarily reflected in continuous surveillance of the vascular barrier and surrounding microenvironment, as well as in the maintenance of a “low-inflammation, high-cleanliness” interface through phagocytosis and clearance. They mediate the removal of apoptotic cells, thereby suppressing secondary inflammatory responses, and can express CD163 to bind hemoglobin-haptoglobin complexes and heme, facilitating the clearance of cellular debris and metabolic waste, reducing oxidative stress-induced damage, and preserving a clean and metabolically stable intracerebral microenvironment (Kim et al., 2006; Uchikawa et al., 2024). In this respect, PvMΦ function both as barrier-adjacent “scavengers” and as key stabilizers that maintain immune quiescence within the perivascular spaces.

Beyond barrier-adjacent clearance, PvMΦ are also closely linked to metabolic waste handling and regulation of vascular responses. They are thought to participate in the processing of aberrant deposits within perivascular spaces, including Aβ-associated burdens, and can influence Aβ-related neurovascular function and disease outcomes (Park et al., 2017). A more interface-specific feature is their capacity to respond to neuronal activity-associated signals: PvMΦ express the purinergic receptor P2Y12, enabling them to sense ATP released during neuronal activity and to modulate local vasodilation through calcium signaling, thereby matching regional blood supply to energy demand and contributing to hemodynamic regulation. Under conditions of metabolic stress or vascular dysfunction, PVM-associated VEGF signaling has been reported to be linked to restoration of endothelial metabolic and transport phenotypes (Császár et al., 2022; Wen et al., 2024). These characteristics indicate that, under homeostatic conditions, PvMΦ not only perform immune clearance but also participate in neurovascular coupling and energy supply-demand matching through “sensing-response” mechanisms.

**Figure 5.**
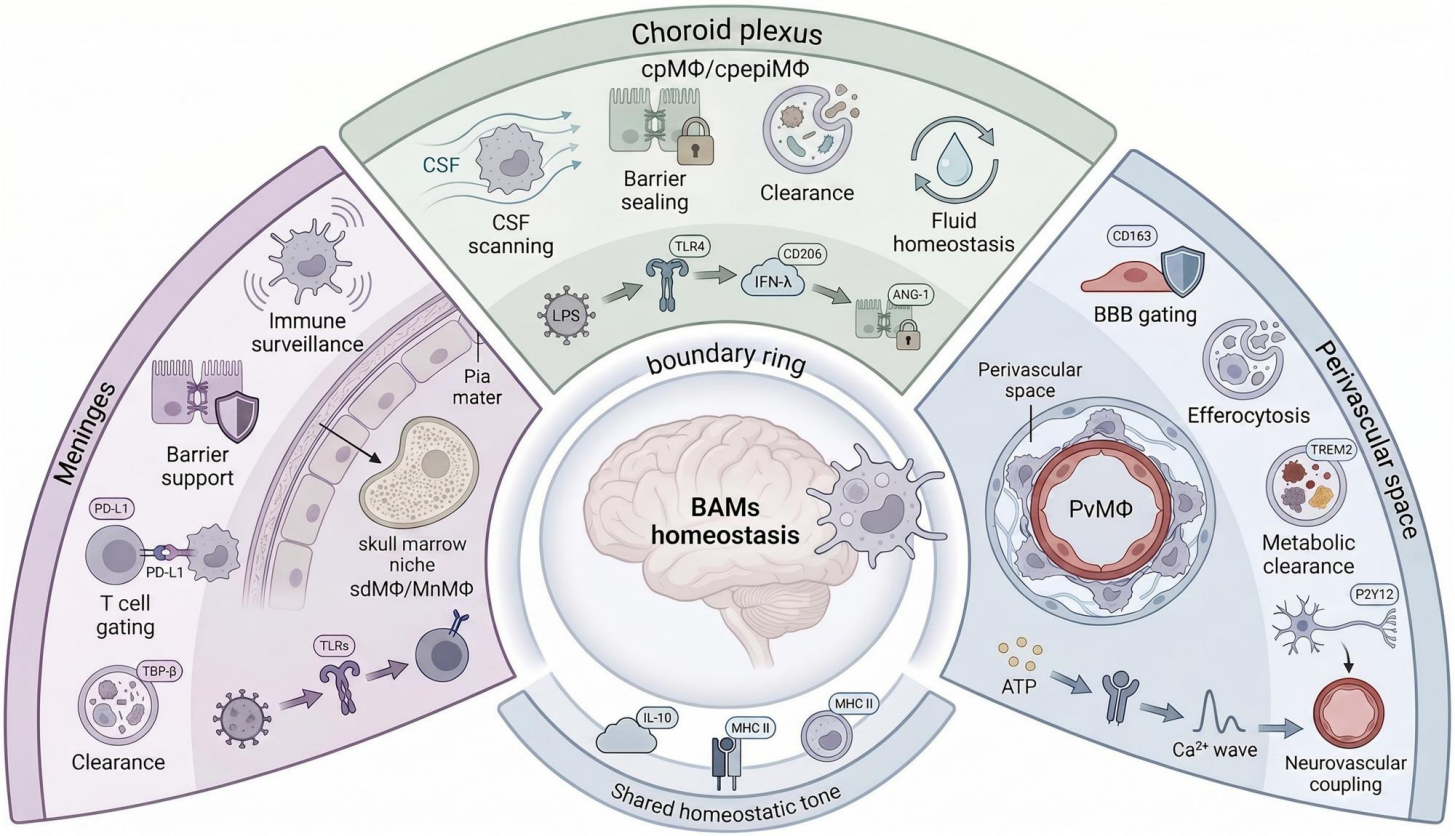
Integrated schematic of homeostatic functions of BAM subsets across the three interface compartments: sdMΦ/MnMΦ in the meninges, PvMΦ in perivascular spaces, and cpMΦ/cpepiMΦ in the choroid plexus.

From the perspective of homeostatic functional axes, the core outputs of PvMΦ can be more reliably summarized as a three-tiered interplay of “clearance and immune quiescence-vascular integrity and metabolic regulation-neurovascular functional modulation.” First, by residing within perivascular spaces and possessing robust phagocytic capacity, PVMs remove cellular debris and pathological loads and restrict local immune activation, thereby supporting immune quiescence and microenvironmental stability. Second, under physiological conditions, PVMs are thought to contribute to the maintenance of vascular integrity and metabolic regulation, while under pathological burdens, such as Aβ-associated stress, they participate in effector pathways that alter neurovascular function. Third, in the presence of vascular risk factors such as hypertension, PVMs can become a significant source of oxidative stress, influencing neurovascular regulation and cognitive outcomes through pathways including NOX2/ROS, highlighting their dual roles along the continuum from homeostatic maintenance to pathological imbalance (Figure 5).

## The Double-Edged Roles of BAMs in CNS Diseases

As immune sentinels residing at CNS border regions, BAMs maintain BBB integrity, clear metabolic waste, and regulate immune tolerance under homeostatic conditions; however, under disease states, dysregulation of these functions can drive neuroinflammation, tissue injury, and disease progression. Notably, the pathological roles of BAMs exhibit pronounced spatiotemporal specificity: the same subset can shift from protective to detrimental functions depending on disease stage, interface compartment, and stimulus intensity, thereby displaying a dual nature in which BAMs act as both guardians and potential drivers of pathology.

### Is: Transition From Acute Injury to Chronic Repair

Following IS, BAMs undergo dynamic transitions between proinflammatory and anti-inflammatory phenotypes, with particularly prominent double-edged effects. Under homeostatic conditions, CD206^+^ BAMs contribute to maintaining vascular integrity, including regulating endothelial cell proliferation and activating ERK1/2 signaling pathways (Wen et al., 2024; Yu et al., 2026). During the acute phase of stroke, however, BAMs rapidly enter an inflammation-amplifying state: the CD206^+^ subset markedly upregulates TNF and other proinflammatory cytokines, chemokines, and genes associated with vascular leakage (including Vegfa, Hif1a, Mmp8, and Mmp12), accompanied by extracellular matrix (ECM) remodeling, and emerges as a major mediator of TNF signaling (Yu et al., 2026). Concurrently, under the regulation of transcription factors such as STAT3, BAMs undergo functional reprogramming, characterized by upregulation of Myc and Nfil3, as well as induction of iNOS expression and inflammation-related genes after ischemic injury, indicating a shift toward a proinflammatory phenotype (Rajan et al., 2020; Yu et al., 2026). Upon entry into the recovery phase, this inflammatory program does not persist linearly; instead, inflammation-associated pathways decline, while programs related to vascular integrity maintenance and repair become progressively enhanced (Bogale et al., 2021; Yu et al., 2026).

During IS, key BAM subsets and their spatial localization exhibit pronounced interface-specific differences, and the contributions of individual compartments are not equivalent. In the acute phase of stroke, BBB disruption is closely associated with ECM and tight junction degradation, with matrix metalloproteinase-9 (MMP-9) degrading components such as collagen IV and laminin, thereby exacerbating barrier permeability abnormalities (Guo et al., 2025). Clinically, peripheral blood levels of MMP-9 correlate with stroke severity and unfavorable outcomes (Ramos-Fernandez et al., 2011). In contrast, meningeal BAMs engage in immunoregulatory interactions during the recovery phase, suppressing activation of infiltrating T cells and reducing the risk of immune-mediated secondary injury (Min et al., 2024). In addition, on post-stroke day 3, CD163^+^ BAMs are enriched for cell cycle-related genes, indicating enhanced proliferative activity and suggesting that BAM population size and activation state undergo substantial temporal changes (Rajan et al., 2020).

From a mechanistic perspective, ischemia-hypoxia and DAMP signals triggered by stroke drive BAMs at CNS borders to sense and amplify inflammatory responses. BAMs express molecules such as TLR4, TRIF, and RIPK1 and participate in DAMP-mediated inflammatory signaling; subsequent release of cytokines and chemokines promotes immune cell infiltration into the brain, which, together with vascular leakage and matrix degradation, leads to BBB breakdown, edema formation, and increased oxidative stress, ultimately exacerbating neuronal injury (Beuker et al., 2022; Yu et al., 2026). However, during the prolonged recovery phase, signaling pathways involving HIF-1α and VEGF may also contribute to tissue repair, indicating that the same hypoxia-responsive axis can exert either detrimental or beneficial effects depending on disease stage (Pedragosa et al., 2018). Therefore, the central challenge regarding BAM function in stroke is not the presence of inflammation per se, but whether the timing, magnitude, and duration of inflammatory responses are appropriately constrained.

### AD: A Critical Imbalance Between Protection and Injury

In AD, BAMs exhibit a characteristic double-edged role in processes surrounding Aβ deposition and clearance. On the protective side, perivascular BAMs/PVMs participate in perivascular Aβ handling and influence cerebral amyloid angiopathy (CAA) burden, suggesting their potential role in perivascular Aβ homeostasis and clearance (Hawkes & McLaurin, 2009). In addition, border compartments together with perivascular and CSF-associated clearance pathways jointly mediate Aβ efflux and CAA regulation, with PVMs/BAMs considered integral cellular components of this interface clearance network (van Veluw et al., 2024). However, this protective cascade may become progressively imbalanced during disease progression, resulting in reduced clearance efficiency, heightened inflammation, and an increased risk of vascular injury.

With respect to key subsets and spatial localization, perivascular BAMs are among the central executors of Aβ-related processes, functioning not only in phagocytic clearance but also potentially in the direct mediation of neurovascular dysfunction (Park et al., 2017). Newly published work in AppNL-F knock-in mice links early glymphatic failure to the loss of parenchymal border macrophages, suggesting that CSF Aβ-related impairment of border macrophage populations may precede prominent parenchymal plaque deposition and contribute to defective waste clearance (Liu et al., 2026). Changes in BAM composition and activation state, including replacement by monocyte-derived cells, may further amplify inflammatory responses and accelerate neuronal damage and cognitive decline (Wu et al., 2021). Accordingly, the spatial continuum linking border compartments, perivascular regions, and CSF pathways provides a critical framework for understanding the dual actions of BAMs in AD.

From a mechanistic perspective, Aβ can be cleared by BAMs and can also trigger BAM-mediated injury signals. Along injurious pathways, binding of Aβ to CD36 on BAM surfaces induces reactive oxygen species (ROS) production, thereby promoting neurovascular dysfunction and cognitive impairment; genetic or functional loss of CD36 suppresses ROS generation and mitigates Aβ-induced neurovascular deficits (Park et al., 2017; Uekawa et al., 2023). At the metabolic level, Aβ/APP-associated cellular models exhibit mitochondrial dysfunction and elevated ROS, while activation of SIRT1/PGC-1α-dependent mitochondrial biogenesis programs improves oxidative phosphorylation-related protein expression and reduces ROS, suggesting a functional link between the PGC-1α axis and Aβ-associated metabolic injury (Yao et al., 2022; Yin et al., 2022).

### MS: A Niche for Immune Attack

In MS, BAMs participate in immune regulation and inflammatory responses and can extend their functional roles from proinflammatory drivers to programs associated with chronic degeneration as the disease progresses (Kamma et al., 2022; Sun & Jiang, 2024). During the early stages of disease, BAMs promote inflammatory responses through antigen presentation and T cell activation, thereby facilitating demyelination; during the chronic progressive phase, BAMs may remain persistently activated, enhancing neurodegenerative processes by upregulating proinflammatory genes and downregulating homeostasis-associated genes, reflecting a dual disease-course impact characterized by “early amplification of immune attack and late maintenance of chronic inflammatory tone” (Montilla et al., 2023).

With respect to key subsets and spatial localization, perivascular BAMs have been strongly implicated in the formation of focal lesions and the exacerbation of demyelination. Studies have shown that, in MS models, BAMs can activate CD4^+^ T cells, thereby intensifying neuroinflammation and demyelination (Montilla et al., 2023). In parallel, perivascular BAMs release GALECTIN-3, which activates the oligodendrocyte apoptotic Caspase-8/p38 MAPK pathway and further aggravates demyelinating pathology (Xue et al., 2023). Collectively, these findings indicate that in MS, BAMs are not merely bystanders of inflammation but actively contribute to disease progression through an “antigen presentation-T cell activation-oligodendrocyte injury” cascade.

From a mechanistic standpoint, MS initiation and progression can be conceptualized as a multistep amplification process driven by border immune activity: BAMs enhance local immune attack via antigen presentation and T cell activation, while simultaneously releasing GALECTIN-3 and engaging oligodendrocyte apoptosis-related pathways to accelerate myelin damage; subsequently, during the chronic phase, they sustain proinflammatory programs and attenuate homeostasis-associated gene expression, thereby promoting neurodegeneration (Montilla et al., 2023; Vara-Pérez & Movahedi, 2025; Xue et al., 2023). Accordingly, the double-edged role of BAMs in MS is better described as an “immune amplifier and chronic inflammation maintainer,” with functional directionality shaped by disease stage and the local microenvironment.

### TBI: Early Protective Inflammation and Secondary Injury

TBI induces rapid activation and redistribution of brain macrophage populations, including BAMs, microglia, and infiltrating monocyte-derived macrophages. A recent study focusing on the BAM-associated marker MRC1/CD206 showed that MRC1 contributes to an early neuroprotective inflammatory response after TBI in mice; MRC1 deficiency was associated with reduced CD68^+^ macrophage/microglial responses and altered neuroinflammatory marker expression, suggesting that early CD206^+^ border-associated macrophage activity may support rather than merely exacerbate post-traumatic immune defense (Strehle et al., 2025). This finding complements earlier evidence that macrophage responses after TBI are heterogeneous and stage dependent, with both reparative and deleterious programs emerging over time (Hsieh et al., 2013).

Mechanistically, TBI provides a useful example of why BAM functions cannot be interpreted simply as proinflammatory or anti-inflammatory. During the early phase, BAM-associated phagocytosis, antigen sensing, and chemokine production may help contain tissue damage and coordinate debris clearance at CNS borders. During later phases, persistent myeloid activation, barrier disruption, and peripheral cell infiltration can sustain secondary injury and neurodegeneration. Thus, TBI further supports a stage-specific model in which BAM activation is potentially protective when transient and spatially constrained, but harmful when prolonged or coupled to uncontrolled immune recruitment.

### Brain Tumors: “Accomplices” Within the Tumor Microenvironment

In brain tumors, BAMs can drive malignant progression by remodeling immunosuppressive microenvironments, promoting angiogenesis, and supporting tumor invasion, with pronounced anatomical location-specific features; however, in certain tumor types, BAMs may also exert tumor-suppressive effects, reflecting a more complex double-edged profile (Akay & Saleh, 2025; Li et al., 2024). Overall, the tumor microenvironment tends to bias BAMs toward immunosuppressive and proinvasive phenotypes, although the directionality of these effects may vary according to tumor type, spatial context, and local microenvironmental signals.

Regarding key subsets and spatial localization, tumor-associated BAMs cooperate with tumor-associated macrophages to establish immunosuppressive niches. In glioblastoma, BAMs and tumor-associated macrophages jointly promote immune evasion (Kienzler & Becher, 2024; Ochocka et al., 2021). Tumor-associated BAMs exhibit high expression of ARG1 and PD-L1, suppressing CD8^+^ T cell antitumor activity by depleting L-arginine in the microenvironment; spatial transcriptomic analyses further reveal close spatial proximity between tumor-associated BAMs and T cells, indicating potent suppression at short range (Liu et al., 2024; Ravi et al., 2022). Recent spatial transcriptomics in glioblastoma patients with radionecrotic changes further identified infiltration of border-associated macrophages in affected tissue and suggested a possible contribution to gliosis, extending the relevance of BAMs from tumor immunosuppression to treatment-related tissue injury and repair responses (Seferbekova et al., 2026). In contrast, in medulloblastoma, BAMs can express CXCL4 to antagonize the CXCL12/CXCR4 axis, thereby inhibiting proliferation and migration of tumor progenitor cells, suggesting that BAMs may also exert antitumor effects in specific tumor contexts (Molina-Arocho & Haldar, 2023; Pokrajac et al., 2023).

From a mechanistic standpoint, the double-edged roles of BAMs in brain tumors can be summarized as two opposing pathways. Along the protumorigenic route, tumor-derived signals recruit and reprogram BAMs toward immunosuppressive phenotypes characterized by ARG1 and PD-L1 expression, enabling local suppression of T cell activity while promoting tumor cell invasion through secretion of matrix metalloproteinases that disrupt tight junctions. In addition, BAMs can release growth factors and cytokines that support tumor cell proliferation, survival, and migration, and their senescence-associated chronic low-grade inflammatory state may further contribute to immune dysfunction and tumor progression (Akay & Saleh, 2025; Pires-Afonso et al., 2020). Accordingly, the functional orientation of BAMs within tumors is more strongly determined by the combined influence of tumor type, spatial localization, and microenvironmental cues.

### Cross-Disease Comparative Summary

**Figure 6.**
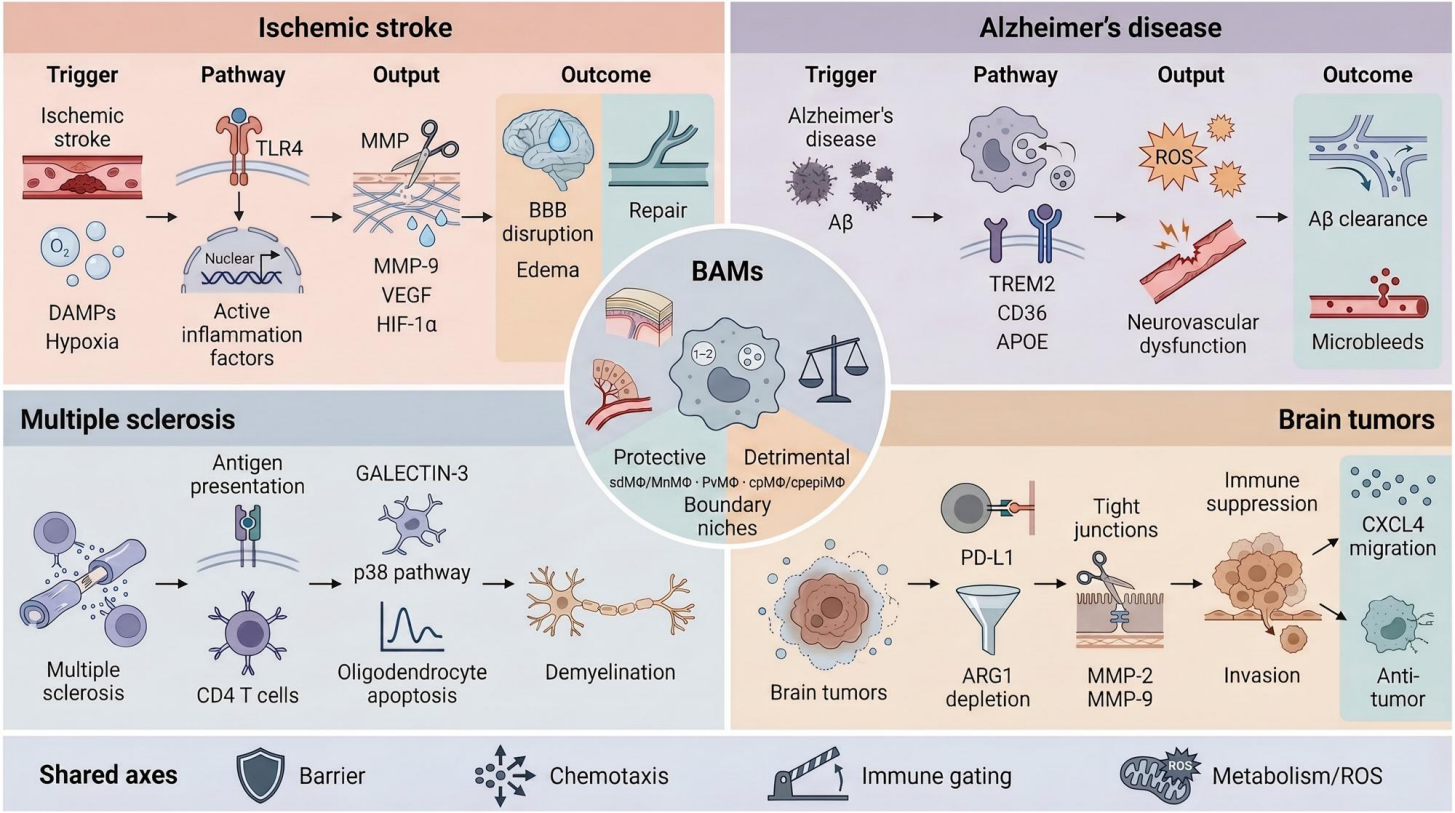
Integrated mechanistic overview of the double-edged roles of BAM subsets across five major CNS disease contexts, including ischemic stroke (IS), Alzheimer’s disease (AD), multiple sclerosis (MS), traumatic brain injury (TBI), and brain tumors.

Overall, BAMs play dual roles across CNS diseases: they can confer protection during early defensive phases but drive injury and disease progression during chronic stages. Their pathological transitions often depend on microenvironmental cues, epigenetic reprogramming, and population remodeling driven by infiltration of bone marrow-derived cells (Fixsen et al., 2023; Gerganova et al., 2022; Pedragosa et al., 2018; Wang et al., 2024). From a cross-disease perspective, IS, AD, MS, TBI, and brain tumors—despite distinct initiating factors—share convergent BAM-centered nodes involving barrier integrity or disruption, chemotactic recruitment, and immune-regulatory threshold setting. Specifically, stroke prominently features MMP-mediated basement membrane degradation and TLR4/NF-κB-driven immune recruitment (Gerganova et al., 2022); AD highlights the coexistence of Aβ clearance failure and CD36-ROS-associated vascular injury (Liu et al., 2026; Uekawa et al., 2023); MS emphasizes antigen presentation, T cell activation, and GALECTIN-3-linked oligodendrocyte damage (Montilla et al., 2023); TBI underscores the need to distinguish early MRC1/CD206-associated protective inflammation from later secondary injury (Strehle et al., 2025); and tumors are characterized by ARG1/PD-L1-mediated immunosuppression, MMP-associated invasion, and BAM infiltration in radionecrotic tissue (Pu & Ji, 2022; Seferbekova et al., 2026) (Figure 6).

Regarding sources of divergence, inconsistencies across studies commonly arise from the combined effects of multiple factors, including differences between animal models and human samples, variation in observation windows (acute, subacute, or chronic), nonuniform cell-definition criteria (marker selection), and methodological disparities among scRNA-seq, spatial omics, imaging, and functional assays that introduce population-specific “capture bias.” For example, the same hypoxia-responsive axis may exert opposing effects during the acute versus recovery phases of stroke, while in AD, strategies aimed at enhancing Aβ clearance must concurrently account for vascular side effects such as microhemorrhage (Park et al., 2017; Taylor et al., 2023). Together, these observations underscore disease stage and spatial context as critical variables for interpreting apparently conflicting findings.

## Future Research Directions for BAMs

As immune sentinels that reside long term at CNS borders, BAMs exhibit pronounced duality and spatiotemporal dependence in both homeostatic maintenance and disease progression, owing to their unique multi-interface localization and plastic transcriptional programs. Current studies have progressively delineated their subset specialization, regulatory networks, and functional outputs across compartments such as the meninges, choroid plexus, and perivascular spaces; nevertheless, critical limitations remain, including model extrapolation, inconsistent definitional criteria, and challenges in cross-species comparability. These gaps underscore the need for more standardized, verifiable, and translatable research frameworks to advance the field.

### Core Consensus: Multi-Interface Localization and Functional Axes of BAMs

A growing consensus holds that BAMs are not a homogeneous population of “border macrophages,” but rather a multi-subset system shaped by distinct interface niches within the meninges, choroid plexus, and perivascular spaces. Their spatial localization determines the sources of signals to which they are chronically exposed, the structural constraints they encounter, and the humoral environments they sample, thereby sculpting differentiated homeostatic functional axes. In general, under homeostatic conditions, BAMs contribute to maintaining a low-inflammatory, highly stable CNS microenvironment through coordinated functions that include immune surveillance and pathogen interception, structural support of barriers and interfaces, clearance of apoptotic cells and metabolic waste, and regulation linked to vascular reactivity and fluid homeostasis. Importantly, these multi-interface niches represent more than positional differences; they define the prioritization of BAM “primary tasks” across compartments. Specifically, the meninges are more oriented toward peripheral signal input and immune-threshold gating, the choroid plexus toward CSF interface monitoring and blood-CSF barrier integrity, and the perivascular spaces toward neurovascular unit gating, metabolic clearance, and neurovascular coupling. Within this framework, BAM-mediated “homeostatic guardianship” and “pathological transformation” can be understood as differential outputs of the same regulatory network operating across distinct interfaces and temporal scales.

### Key Controversies: Definitional Criteria, Subset Boundaries, Spatiotemporal Dependence, and Model Differences

It should be emphasized that substantial limitations remain in the current understanding of BAMs. First, most existing studies rely on inbred mouse models maintained under highly controlled, pathogen-free conditions, which may not adequately capture the true dynamics of immune cells under human disease states; moreover, marked differences likely exist between human and murine BAMs in terms of cellular complexity and renewal kinetics. In humans, the rules governing BAM turnover remain poorly defined, whereas increased monocyte infiltration has been observed in aged mice; whether a corresponding phenomenon occurs during human aging and how it relates to neurodegenerative disease remain to be established. Second, the lack of uniformity across laboratories in BAM definitions and isolation strategies—including marker selection and tissue compartmentalization—directly compromises cross-study comparability and amplifies apparent discrepancies among reported conclusions. Third, tissue macrophages may lose key aspects of their specialized identity during *in vitro* culture, introducing systematic bias between *in vitro* functional assays and authentic *in vivo* states. Finally, although animal models often show a relatively low degree of replacement of embryonically derived BAMs by monocyte-derived cells, this proportion may differ substantially in humans under conditions of aging or inflammation; such uncertainty complicates the interpretation of resident versus replenishment mechanisms and the contribution of distinct cellular origins in disease. Therefore, the core of the current controversy is not simply whether a given conclusion is correct or incorrect, but rather that BAM identity, boundaries, and dynamics cannot be uniformly delineated when studies differ in cell-definition criteria, temporal windows, spatial context, and model systems.

### Outlook: Standardization, Cross-Species Mapping, Spatial Multi-Omics, and Targeted Intervention and Delivery Strategies

To achieve substantive advances in both mechanistic understanding and translational application, future research must first establish a shared foundation of standardization and comparability. On the one hand, this requires unified frameworks for defining and subclassifying BAMs across compartments—integrating spatial localization, combinatorial marker panels, and transcriptional modules—while adopting reproducible isolation procedures and data-analysis pipelines whenever possible. On the other hand, it is essential to delineate the developmental origins, adult maintenance, and replenishment mechanisms of distinct BAM subsets, including whether monocyte-derived supplementation becomes more prominent in humans under aging or inflammatory conditions. Second, cross-species mapping should move beyond superficial gene-name similarity toward homology at the levels of cellular subsets and functional circuits. Third, spatial multi-omics will be indispensable for resolving how interface niches dictate function, particularly at newly characterized border structures such as ACE points, DALT, SLYM, and skull-dural channels. These structures may define previously underappreciated routes for CSF drainage, antigen sampling, immune-cell trafficking, and skull marrow-CNS crosstalk, and should therefore be incorporated into future BAM mapping studies. At the same time, interactions between BAMs and microglia, astrocytes, endothelial cells, stromal cells, lymphocytes, and peripheral myeloid cells remain critical knowledge gaps. Finally, translational strategies targeting specific BAM subsets warrant focused development. Approaches based on gene editing, nanocarrier-mediated targeted delivery, and small-molecule modulators should be designed in a compartment-, subset-, and stage-specific manner to avoid indiscriminate suppression that disrupts homeostasis or excessive enhancement of immune clearance that provokes barrier-related side effects.

## Data Availability

All data generated or analyzed during this study are included in this article and/or its supplementary material files. Further enquiries can be directed to the corresponding author.
